# Biophysical and structural study of La Crosse virus endonuclease inhibition for the development of new antiviral options

**DOI:** 10.1107/S205225252400304X

**Published:** 2024-04-24

**Authors:** Mikael Feracci, Sergio Hernandez, Laura Garlatti, Clemence Mondielli, Renaud Vincentelli, Bruno Canard, Juan Reguera, François Ferron, Karine Alvarez

**Affiliations:** a Université Aix-Marseille, Architecture et Fonction des Macromolécules Biologiques (AFMB)–UMR7257 CNRS–Case 932, 163 Avenue de Luminy, 13288 Marseille CEDEX 09, France; b Université Lille; INSERM, UMR-S 1172, Lille Neuroscience and Cognition Research Centre, 59000 Lille, France; c OmegaChem, Lévis, 480 Rue Perreault, Québec G6W 7V6, Canada; d Evotec (France) SAS, Campus Curie, 195 Route d’Espagne, 31036 Toulouse, France; e European Virus Bioinformatics Center, Leutragraben 1, 07743 Jena, Germany; Chinese Academy of Sciences, China

**Keywords:** *Bunyavirales*, orthobunyaviruses, endonuclease inhibitors, La Crosse virus, metal chelators

## Abstract

The large *Bunyavirales* order includes several families of viruses with a segmented ambisense (−) RNA genome and a cytoplasmic life cycle that starts by synthesizing viral mRNA. The initiation of transcription, which is common to all members, relies on an endonuclease activity that is responsible for cap-snatching. In La Crosse virus, an orthobunyavirus, it has previously been shown that the cap-snatching endonuclease resides in the N-terminal domain of the L protein. Orthobunyaviruses are transmitted by arthropods and cause diseases in cattle. However, California encephalitis virus, La Crosse virus and Jamestown Canyon virus are North American species that can cause encephalitis in humans. No vaccines or antiviral drugs are available. In this study, three known Influenza virus endonuclease inhibitors (DPBA, L-742,001 and baloxavir) were repurposed on the La Crosse virus endonuclease. Their inhibition was evaluated by fluorescence resonance energy transfer and their mode of binding was then assessed by differential scanning fluorimetry and microscale thermophoresis. Finally, two crystallographic structures were obtained in complex with L-742,001 and baloxavir, providing access to the structural determinants of inhibition and offering key information for the further development of *Bunyavirales* endonuclease inhibitors.

## Abstract 2

A structural analysis of several metal-ion binders that inhibit viral endonucleases is performed.

## 1. Introduction

Orthobunyaviruses belong to the *Peribunyaviridae*, a family in the *Bunyavirales* order that harbours several deadly pathogens. These viruses form a serologically and genetically related group; they are arthropod-borne and some have been shown to cause neuroinvasive diseases in humans, which primarily occur in children. La Crosse virus (LACV) is the most common neuroinvasive arboviral infection in children (Boutzoukas *et al.*, 2023 ▸) in the United States (Vahey *et al.*, 2021 ▸) and serves as a study model for other neuroinvasive ortho­bunyaviruses such as Jamestown Canyon orthobunyavirus (JCV) and Snowshoe hare orthobunyavirus (SSHV) in the USA and Canada (Evans & Peterson, 2021 ▸), Tahyna orthobunyavirus (TAHV), which is widely distributed throughout Europe, Africa and Asia, and Inkoo orthobunyavirus (INKV), which is found in Scandinavia (Lwande *et al.*, 2017 ▸; Vapalahti *et al.*, 1996 ▸). Other animal-infecting orthobunyaviruses include Schmallenberg virus (SBV) and Akabane virus, which severely affect ruminants (Wernike & Beer, 2017 ▸; Yanase *et al.*, 2020 ▸). Reports of the newly identified Cristoli ortho­bunyavirus and the emergence of Umbre orthobunyavirus as agents responsible for fatal encephalitis (Rodriguez *et al.*, 2020 ▸; Pérot *et al.*, 2021 ▸) stress the importance and necesssity of monitoring the impact of orthobunyaviruses on human health and activities. While vaccines have been developed against several animal-infecting orthobunyaviruses, including SBV (Wernike *et al.*, 2022 ▸), no vaccines or therapies are available for those infecting humans. As climate change drives disease-spreading arthropods into new territories (McDermott, 2022 ▸), it is clear that orthobunyaviruses, among other arboviruses, pose a serious threat, with an urgent need for new therapeutic strategies in terms of both vaccines and the development of antivirals.

*Bunyavirales* exhibits a distinctive genomic architecture characterized by a segmented ambisense negative single-stranded RNA genome. The viral RNA (vRNA) segments intricately associate with a specialized replication complex known as the ribonucleoprotein (RNP) complex. The RNP complex plays a pivotal role in safeguarding and processing the vRNA. Structurally, the RNP complex consists of a polymer of nucleoproteins enveloping the vRNA and associates with a single large (L) protein. The L protein serves as the primary effector, showcasing its multifunctional nature by encompassing both endonuclease (EndoN) activity in its N-terminus and RNA-dependent RNA polymerase (RdRp) activity. Viruses belonging to this order have a cytoplasmic life cycle that starts with the synthesis of viral messenger RNA (vmRNA). They use a particular mechanism named cap-snatching to initiate the transcription of vmRNA, and this process is carried out by the multifunctional L protein. During cap-snatching short capped primers derived by endonucleolytic cleavage of host mRNAs by the EndoN are used by the RdRp to initiate the synthesis of vmRNA.

The determined atomic structures of different EndoNs show that they harbour a conserved PD…D/E*x*K catalytic motif in the active site and bind divalent metal ions (Mn^2+^ or Mg^2+^) that are critical for catalytic activity. Thus, the metal-dependent endonuclease (Horst *et al.*, 2019 ▸; Crépin *et al.*, 2010 ▸) cleaves the 5′-end-capped structure of host mRNAs to generate primers for the viral polymerase. The reaction follows a two-metal-ion catalytic mechanism (Morin *et al.*, 2010 ▸; Steitz & Steitz, 1993 ▸). The critical function carried by this domain and its fairly conserved regions make EndoN a suitable target for antiviral development (Saez-Ayala *et al.*, 2018 ▸, 2019 ▸), and EndoN inhibitors that anchor into the active site through metal ions could efficiently block the enzymatic activity and prevent viral transcription (DuBois *et al.*, 2012 ▸; Ferron *et al.*, 2012 ▸).

The concept of antiviral agents targeting metalloenzymes with metal-chelating inhibitors emerged in 1979 and has been validated as an important strategy in drug design (Rogolino *et al.*, 2012 ▸). The development of baloxavir as a chelating viral EndoN inhibitor (Jones *et al.*, 2016 ▸; Noshi *et al.*, 2018 ▸) is one of the success stories in treating Influenza virus (FLUV) infections. Baloxavir marboxil was approved in 2018 in Japan and the USA for the treatment of FLUAV and FLUBV and has been commercialized under the name Xofluza. The first class of FLUV EndoN inhibitors (Tomassini *et al.*, 1994 ▸; Hastings *et al.*, 1996 ▸) were 4-substituted-2,4-dioxobutanoic acids carrying an aromatic scaffold and a chelating diketo-acid (DKA) motif, consisting of γ-ketone, enolizable α-ketone and carboxylic acid moieties, that is able to chelate divalent metal ions (Mg^2+^ or Mn^2+^). Among the DKA series, 2,4-dioxo-4-phenylbutanoic acid (DPBA; phenyl-DKA; Tomassini *et al.*, 1994 ▸; Hastings *et al.*, 1996 ▸; Kowalinski *et al.*, 2012 ▸; DuBois *et al.*, 2012 ▸; Reguera *et al.*, 2010 ▸) has been extensively studied and L-742,001 (piperidinyl-DKA; Hastings *et al.*, 1996 ▸; Kowalinski *et al.*, 2012 ▸; DuBois *et al.*, 2012 ▸) has been identified as a potent inhibitor in both animal model and cell-based antiviral assays (Hastings *et al.*, 1996 ▸). The metal chelation of DPBA and L-742,001 in the FLUV EndoN active site has been deciphered (Kowalinski *et al.*, 2012 ▸; DuBois *et al.*, 2012 ▸; Reguera *et al.*, 2010 ▸), indicating the strength of metal anchoring and the plasticity of the active site responsible for the induced-fit mode of ligand binding. In contrast, baloxavir has been described as a tight-binding inhibitor (Omoto *et al.*, 2018 ▸; Todd *et al.*, 2021 ▸), partly explaining its high anti-influenza efficiency (Jones *et al.*, 2016 ▸; Noshi *et al.*, 2018 ▸).

The potency of DPBA, phenyl-DKA analogs, L-742,001 and piperidinyl-DKA analogs extended to the homologous EndoN domains from Lymphocytic choriomeningitis virus (LCMV; Reguera *et al.*, 2016 ▸; Saez-Ayala *et al.*, 2018 ▸, 2019 ▸), LACV (Reguera *et al.*, 2016 ▸; Fernández-García *et al.*, 2020 ▸), Andes orthohantavirus (ANDV; Fernández-García *et al.*, 2020 ▸), Rift Valley fever virus (RVFV; Fernández-García *et al.*, 2020 ▸), Ebinur Lake virus (EBIV; Kuang *et al.*, 2022 ▸) and Toscana virus (TOSV; Jones *et al.*, 2019 ▸). In previous structural studies, co-structures of DPBA with LCMV EndoN (Saez-Ayala *et al.*, 2018 ▸; PDB entry 5ltn), LACV EndoN (Reguera *et al.*, 2010 ▸; PDB entry 2xi7) and TOSV EndoN (Jones *et al.*, 2019 ▸; PDB entry 6qw5) and that of L-742,001 in complex with LCMV EndoN (Saez-Ayala *et al.*, 2018 ▸; PDB entry 5t2t) demonstrated their induced-fit ligand-binding mode. Imperfect stacking and a lack of polar interactions are partly responsible for residual flexibility and suboptimal potency, as observed for FLUV EndoN. Despite this, some compounds have shown promising inhibition (Fernández-García *et al.*, 2020 ▸), in particular the piperidinyl-DKA analogs that we designed to strengthen the hydrophobic interactions in a putative identified subcavity and on surfaces (conserved regions) of the active site of LCMV EndoN (Mondielli, 2018 ▸).

Because of the homology between *Bunyavirales* and FLUV EndoN domains and the similar binding behaviour of phenyl-DKAs and piperidinyl-DKAs in the EndoN active site, we decided to repurpose baloxavir on a *Bunyavirales* EndoN (LACV) and explore its potential compared with DPBA and L-742,001. By understanding their binding modes, we aimed to propose structural key information for the design of more potent inhibitors targeting EndoNs within the *Bunyavirales* order.

## 2. Materials and methods

### 2.1. Protein expression and purification

cDNA of LACV EndoN (UniProt accession code A5HC98) coding for the N-terminal 183 residues was used to express the protein in *Escherichia coli* pLysS strain cells in Terrific Broth medium supplemented with 34 µ*M* chloramphenicol and 50 µ*M* kanamycin at 18°C overnight after induction with 0.2 m*M* isopropyl β-d-1-thiogalactopyranoide. The cells were disrupted by sonication on ice in lysis buffer (20 m*M* Tris–HCl pH 7.6, 150 m*M* NaCl, 10 m*M* imidazole, 2.5 m*M* β-mercaptoethanol). The protein from the soluble fraction was loaded onto 5 ml nickel resin, washed five times with lysis buffer with 10 m*M* imidazole and five times with lysis buffer with 50 m*M* imidazole, and eluted with four volumes of lysis buffer with 400 m*M* imidazole. The histidine tag of the eluted protein was cleaved using Tobacco etch virus protease at 4°C overnight in dialysis buffer (20 m*M* Tris–HCl pH 7.6, 50 m*M* NaCl, 2.5 m*M* β-mercapto­ethanol). A second affinity-chromatography step was performed to remove uncleaved protein before an anion-exchange purification step. The protein was loaded onto the column (Cytiva RESOURCE Q, 6 ml), washed with 20 volumes of dialysis buffer and eluted with a concentration gradient of NaCl to 1 *M*. The eluted protein was concentrated and loaded onto a Cytiva HiLoad 16/600 Superdex 75 pg column with 20 m*M* HEPES pH 7.6, 150 m*M* NaCl, 2.5 m*M* β-mercaptoethanol for a final purification step. The protein was concentrated to 5–15 mg ml^−1^ depending on its use.

### 2.2. Characterization of compounds and substrate

2,4-Dioxo-4-phenylbutanoic acid (DPBA) and (*Z*)-4-{1-benzyl-4-[(4-chlorophenyl)methyl]-piperidin-4-yl}-2-hydroxy-4-oxobut-2-enoic acid (L-742,001) were synthesized by Dr C. Mondielli (Mondielli, 2018 ▸). (*R*)-12-((*S*)-7,8-Difluoro-6,11-dihydrodibenzo[b,e]thiepin-11-yl)-7-hydroxy-3,4,12,12a-tetrahydro-1H-[1,4]oxazino[3,4-c]pyrido[2,1-f][1,2,4]triazine-6,8-dione (baloxavir) was purchased from MedChemTronica (Sweden). The 19-mer RNA 5′-AUU UUG UUU UUA AUA UUU C-3′ containing a 5′ fluorescent probe (6-FAM) and a 3′ quencher (BHQ1) was purchased from Microsynth AG (Switzerland).

#### 2.2.1. *In vitro* endonuclease fluorescence resonance energy transfer (FRET) assay

An *in vitro* endonuclease assay was used to investigate the inhibition of LACV EndoN by DPBA, L-742,001 and baloxavir. The reaction was conducted using a 20 µl sample in 20 m*M* Tris pH 7.6, 150 m*M* NaCl, 0.01% Tween 20, 1 m*M* DTT and was supplemented with 1 m*M* freshly prepared MnCl_2_. The 19-mer RNA at a concentration of 50 n*M* and a gradient concentration of inhibitor (from 0.004 to 100 µ*M*) were pre-incubated at 25°C in the reaction buffer. The reaction was started upon the addition of LACV EndoN (pre-mixed with 1 m*M* MnCl_2_ and pre-incubated at 25°C for 5 min) at a final concentration of 0.25 µ*M*. Fluorescence upon RNA cleavage was followed in a PHERAstar FSX microplate reader (BMG Labtech, Germany) at 25°C using wavelengths of 465 nm for excitation and 520 nm for emission.

#### 2.2.2. Differential scanning fluorimetry (DSF)

The influence of compound binding on the stability of LACV EndoN was measured by a Thermofluor assay using a CFX Connect Bio-Rad real-time PCR machine at a protein concentration of 5 µ*M* in a buffer consisting of 20 m*M* Tris–HCl pH 7.6, 150 m*M* NaCl, 2.5 m*M* β-mercaptoethanol supplemented with 0.5 m*M* freshly prepared MnCl_2_. The compound concentration was fixed at 50 µ*M* (5% DMSO). In 96-well thin-walled PCR plates, 1 µl inhibitor was added to 17 µl buffer followed by the addition of 2 µl protein. Finally, 2 µl of the fluorescent dye SYPRO Orange (Thermo Fisher, catalog No. 4461146) was added (a fivefold final concentration). Controls were performed using the same protocol without MnCl_2_. The melting-temperature (*T*_m_) values given are the average and standard deviation of three independent experiments.

#### 2.2.3. Microscale thermophoresis (MST)

MST experiments were performed on a Monolith NT.115 instrument (NanoTemper Technologies). The LACV EndoN protein was labelled with the red fluorescent dye NT-647 using the Protein Labelling Kit RED NHS (NanoTemper Technologies). The concentration of the labelled protein was kept constant (at 50 or 100 n*M*), while the ligand concentration was varied. Serial dilution series beginning at 100, 50 or 5 µ*M* for DPBA, L-742,001 and baloxavir, respectively, yielded 16 different concentrations. Experiments were performed in 10 m*M* HEPES buffer pH 7.4, 150 m*M* NaCl, 1 m*M* DTT, 0.05%(*w*/*v*) Tween 20 and were supplemented with 0.5 m*M* freshly prepared MnCl_2_. The final samples contained 5% DMSO to ensure solubility of the compounds. The samples were loaded into standard treated MST-grade glass capillaries (MO-K022, NanoTemper Technologies). After a 5 min incubation period the MST was measured with 70% LED power and 20% infra-red laser power. Controls were performed by following the same protocol in the absence of MnCl_2_. *K*_d_ values were determined using the NanoTemper *MO.Affinity Analysis* software version 2.2.4 and are the average and standard deviation of three independent experiments.

### 2.3. Crystallization, data collection and structure determination

#### 2.3.1. Crystallization

All crystals were grown in 24-well Linbro plates following a previously described protocol (Reguera *et al.*, 2010 ▸). Data sets for the L-742,001–LACV EndoN and baloxavir–LACV EndoN complexes were obtained after soaking the native crystals for 48 h in reservoir buffer supplemented with 5 m*M* MnCl_2_ and 1 m*M* L-742,001 or baloxavir (5% DMSO). The crystals were flash-cooled in liquid nitrogen in reservoir buffer supplemented with 5 m*M* MnCl_2_, 1 m*M* L-742,001 or baloxavir (5% DMSO) and 30% glycerol.

#### 2.3.2. Data collection and structure determination

Data sets for LACV EndoN in complex with L-742,001 or baloxavir were collected on the PROXIMA-1 and PROXIMA-2 beamlines at the SOLEIL synchrotron, Gif-sur-Yvette, France. All data sets, from image processing to model refinement, were handled using the *CCP*4 suite (Agirre *et al.*, 2023 ▸). The data were processed, analysed and scaled using the *xia*2/*DIALS* package (Beilsten-Edmands *et al.*, 2020 ▸; Winter, 2010 ▸; Winter *et al.*, 2018 ▸). The phase of LACV EndoN in complex with L-742,001 was obtained using *Phaser* (McCoy *et al.*, 2007 ▸) using PDB entry 2xi7 as a model. Structure handling and refinement were performed using *Coot* and *REFMAC*5, respectively (Emsley *et al.*, 2010 ▸; Murshudov *et al.*, 2011 ▸). Two extra densities in the active site of each chain, corresponding to manganese ions, were observed. After the addition of these and refinement, a well shaped extra density confirmed the presence of L-742,001 in the active site of the four protein molecules. Upon addition of these four molecules with *Coot*, refinement was performed. The structure of LACV EndoN in complex with baloxavir was obtained using the previously solved structure following the same workflow. Extra density was observed for two manganese ions in the active site of each of the four molecules in the asymmetric unit, and after the addition of these and refinement four extra densities were observed for baloxavir, but only three were built. The last density, which was less well determined, showed a possible low occupancy, with the section from Tyr49 to Phe55 having two alternative positions. Data-collection and refinement statistics are given in Table 1 ▸. Structure analysis was performed using *PyMOL* (version 2.5.5; Schrödinger) and *LigPlot* (Wallace *et al.*, 1995 ▸; Laskowski & Swindells, 2011 ▸).

## 3. Results and discussion

### 3.1. Evaluation of DPBA, L-742,001 and baloxavir for the inhibition of LACV EndoN

DPBA, L-742,001 and baloxavir are three known inhibitors of the FLUV EndoN activity, which is located in the N-terminal half of the polymerase acidic (PA) subunit (PA-N_ter_; Kowalinski *et al.*, 2012 ▸; Song *et al.*, 2016 ▸; Omoto *et al.*, 2018 ▸). Baloxavir has been an FDA-approved drug for the treatment of uncomplicated flu since 2018 as its prodrug form baloxavir marboxil (O’Hanlon & Shaw, 2019 ▸). The metal-ion chelating motifs of these inhibitors allow them to bind to the PA-N_ter_ active site. The interaction of DPBA and L-742,001 through their DKA moiety has already been documented in LACV, LCMV, TOSV and EBIV EndoNs (Reguera *et al.*, 2010 ▸; Saez-Ayala *et al.*, 2018 ▸; Jones *et al.*, 2019 ▸; Kuang *et al.*, 2022 ▸).

#### 3.1.1. Evaluation of DPBA, L-742,001 and baloxavir in an *in vitro* LACV endonuclease activity assay

The inhibition efficacies of the three ligands were tested against LACV EndoN in an endonuclease FRET assay and the results are summarized in Table 2 ▸. This was performed following the cleavage of a 19-mer RNA labelled with a 5′ fluorescent probe (6-FAM) and a 3′ quencher (BHQ1) in the presence of a range of ligand concentrations upon the addition of 0.25 µ*M* LACV EndoN. In this FRET inhibition assay, the order in which the three partners of the catalytic complex (protein, ions and substrate RNA) are introduced is decisive. The reaction is triggered by adding the protein pre-incubated with ions to a mixture containing substrate RNA, ions and ligand. The design of the assay may constitute a limitation to the complete assessment of the binding efficiency of baloxavir to the enzyme, given the competition between substrate RNA, free ions and ligand. As described for FLUV, baloxavir is a tight-binding inhibitor with slow dissociation and acts by preventing binding of the RNA substrate (Omoto *et al.*, 2018 ▸; Todd *et al.*, 2021 ▸). In the context of the current biochemical assay, the interplay between the RNA substrate and the ligand presents an unfavourable scenario for binding of the metal-chelating ligand. Nevertheless, DPBA, L-742,001 and baloxavir exhibit a noteworthy submicromolar inhibitory efficacy, showing IC_50_ values of 0.99, 0.65 and 0.34 µ*M*, respectively. Intriguingly, the inhibitory magnitudes are remarkably comparable, with L-742,001 and baloxavir demonstrating only a 1.5-fold and a threefold greater efficiency than DPBA, respectively. This unexpected uniformity of the inhibition values underscores the remarkable potency of these compounds in the face of a challenging biochemical landscape.

#### 3.1.2. Evaluation of thermal stability by DSF

**Figure 1:**
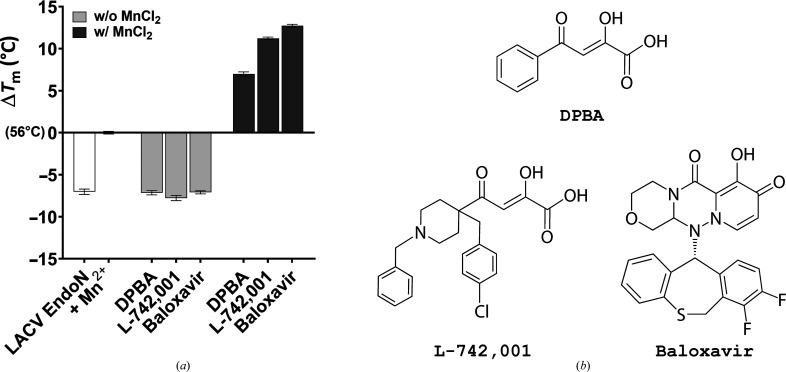
(*a*) DSF experiments showing the stabilizing effect of different ligands on LACV EndoN in the presence or absence of manganese. The values are reported as Δ*T*_m_ using LACV EndoN in the presence of manganese as a baseline (*T*_m_ ≃ 56°C). (*c*) Structures of the studied ligands.

DSF is used to assess protein stabilization or destabilization upon the addition of cofactors by measuring the thermal stability (the thermal melting transition *T*_m_) arising from interaction with a fluorescent dye (SYPRO Orange) during denaturation. The addition of any cofactor (*i.e.* metal ions) or compounds that interact with the protein results in a modification of its *T*_m_. DSF was used to assess the interaction of DPBA, L-742,001 and baloxavir with LACV EndoN; the results are presented in Fig. 1 ▸ and are summarized in Table 2 ▸. Without ions, LACV EndoN has a *T*_m_ of 49°C. Upon the addition of 0.5 m*M* MnCl_2_, its *T*_m_ increases by ∼7°C, showing a strong stabilizing effect by the metal ions. Upon the addition of DPBA, L-742,001 and baloxavir additional thermal melting transition increases of ∼7, ∼11 and ∼13°C, respectively, are observed as a consequence of ligand binding. Without metal ions, no protein stabilization is observed in the presence of ligands, highlighting the absence of interaction with LACV EndoN. As observed by Reguera and coworkers in the crystalline structure of the LACV EndoN–DPBA complex, binding of DPBA is only achieved via the DKA motif (Reguera *et al.*, 2010 ▸). This interaction via the Mn1 and Mn2 ions is sufficient to generate a potent protein stabilization motif (Reguera *et al.*, 2010 ▸). The greater stabilization of LACV EndoN in the presence of L-742,001 (+4°C) or baloxavir (+6°C) compared with DPBA indicates that additional contacts are made, making these two compounds even better ligands. We hypothesized that unlike DPBA, which is too small to establish contacts with the amino acids of the LACV EndoN active site, L-742,001 and baloxavir offer more favourable structural characteristics for establishing contacts. The flexibility of the two wings of L-742,001 and the compactness of baloxavir, despite its size, may be essential elements in their binding in the rather flat LACV EndoN active site.

#### 3.1.3. Evaluation of binding affinity by MST

**Figure 2:**
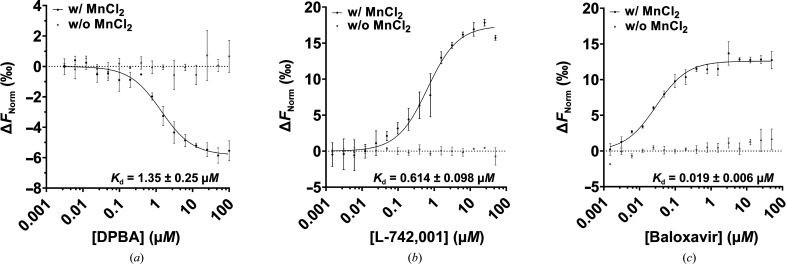
Determination of the dissociation constants (*K*_d_) of LACV EndoN with DPBA (*a*), L-742,001 (*b*) and baloxavir (*c*). *K*_d_ measurement of ligands was performed in the presence or absence of Mn^2+^ ions (*n* = 3).

MST makes use of the thermophoretic movement of a protein in the presence of a ligand to determine the binding affinity. MST was used as an orthogonal method to assess the dissociation constants (*K*_d_) of DPBA, L-742,001 and baloxavir for LACV EndoN; the results are gathered in Fig. 2 ▸ and summarized in Table 2 ▸. LACV EndoN displays a high affinity for the three ligands. For DPBA, a *K*_d_ value of 1.35 µ*M* was measured, which is in a similar range as reported for FLUV PA-N_ter_ (4.5 µ*M*; DuBois *et al.*, 2012 ▸) and LCMV EndoN (5.4 µ*M*; Saez-Ayala *et al.*, 2018 ▸, 2019 ▸). L-742,001 and baloxavir displayed twofold and 68-fold better affinities (*K*_d_ of 0.6 and 0.019 µ*M*, respectively) than DPBA. As expected, binding was completely lost in the absence of metal ions, indicating once again that it is mediated by metal-ion chelation. Surprisingly, compared with thermal stability data, while the difference in affinity between DPBA and baloxavir is very pronounced (68-fold), it is much less so between DPBA and L-742,001 (twofold), suggesting a more robust binding mode for baloxavir. In any case, we have shown here that ligand binding is mainly mediated in the active site through the metal-ion trapping efficiency, which could consequently drive the accommodation of the compound (Table 2 ▸).

### 3.2. LACV EndoN complex structures

In order to decipher the structural basis for the binding and inhibition of LACV EndoN by L-742,001 and baloxavir, we performed soaking experiments using the crystallogenesis conditions described by Reguera *et al.* (2010 ▸). There are very few structures of LACV EndoN, either apo or in complex with ligands. Those obtained by Reguera *et al.* (2010 ▸) in complex with manganese ions and DPBA highlight the binding mode of DKA metal chelators via keto–enol and carboxylic acid moieties.

#### 3.2.1. L-742,001–LACV EndoN complex

**Figure 3:**
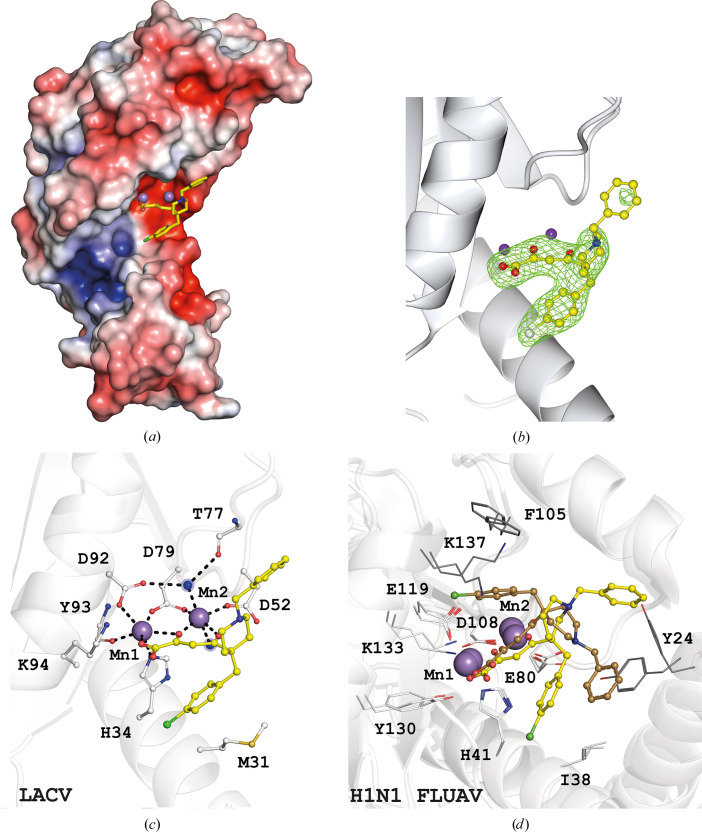
(*a*, *b*, *c*) Crystal structure of LACV EndoN in complex with L-742,001. (*a*) Representation of the electrostatic surface potential of LACV EndoN; the compound is represented as sticks. Mn^2+^ ions are represented as purple spheres. (*b*) An *F*_o_ − *F*_c_ OMIT map corresponding to the ligand (1.5σ). (*c*) Enlargement of the catalytic site showing the coordination of waters, ions and catalytic residues by the ligand. (*d*) Enlargement of the catalytic site of H1N1 FLUAV PA-N_ter_ in complex with L-742,001 (PDB entries 4awg and 5cgv).

The LACV EndoN crystals used in the inhibitor-soaking step were grown in the presence of 5 m*M* MnCl_2_. Native crystals were soaked in a solution consisting of 5 m*M* MnCl_2_ and 1 m*M* L-742,001 with 5% DMSO (final concentration) for 48 h. The collected data set was processed at a resolution of 2.9 Å in space group *P*6_1_22 and the structure was refined to an *R* factor and *R*_free_ of 0.20 and 0.24, respectively, with an average *B* factor of 56 Å^2^ [Fig. 3 ▸(*a*)]. Two manganese ions, Mn1 and Mn2, show full occupancy, with average *B* factors of 43 and 49 Å^2^, respectively, in all four protein molecules in the asymmetric unit. Mn1 is coordinated by the side chains of His34, Asp79 and Asp92 and the carboxylic group of the Tyr93 main chain, while Mn2 is coordinated by the side chains of Asp52 and Asp79 and two structural water molecules. The DKA moiety of L-742,001 completes the octahedral coordination of the two manganese ions [Fig. 3 ▸(*c*)].

The L-742,001 structure can be decomposed into three components: the DKA metal-chelation part described above, a wing consisting of a *p*-chlorobenzyl group and a second wing consisting of a piperidinyl *N*-substituted by a benzyl group. The *p*-chlorobenzyl group is locked between the DKA and the side chains of His34 and Met31 through potential stacking. The DKA and the *p*-chlorobenzyl group of the ligand adopt the same conformation in the active sites of the four molecules.

The *F*_o_ − *F*_c_ map of the benzyl-piperidinyl wing of all four molecules is poorly defined [Fig. 3 ▸(*b*)] due to a possible rotation of the benzyl ring that does not make direct contact with the residues of the active site, and which is mainly exposed to the solvent. No protein residues interfere with the rotation of the benzyl group around the C—N bond axis [Fig. 3 ▸(*c*)].

Compared with the L-742,001–H1N1 FLUAV PA-N_ter_ complex [Fig. 3 ▸(*d*)] the DKA motif is positioned similarly, as expected, with an ion-distribution system that is completely conserved between the two viruses. On the other hand, the active site of FLUAV PA-N_ter_, which is smaller and more constrained, seems to move to allow accommodation of the compound in at least two positions. One is very similar to that in LACV EndoN, mediated by the locking of the *p*-chloro­benzyl group between His41 and Ile38, and the other is mediated by the stacking of the benzyl moiety with the flexible Tyr24, which is absent in the LACV EndoN active site.

These results show that despite the structural identity of LACV EndoN and FLUAV PA-N_ter_, it is not possible to perform hit-to-lead optimization on the basis of the FLUAV model and that the complex obtained with LACV EndoN offers more solid data for efficient drug design.

#### 3.2.2. Baloxavir–LACV EndoN complex

**Figure 4:**
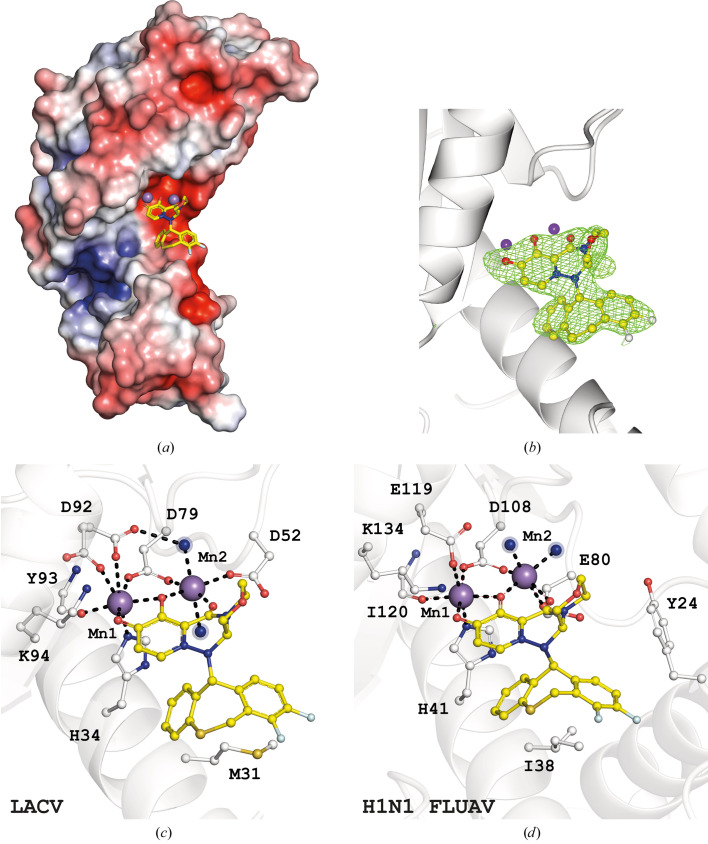
(*a*, *b*, *c*) Crystal structure of LACV EndoN in complex with baloxavir . (*a*) Representation of the electrostatic surface potential of LACV EndoN; the compound is represented as sticks. Mn^2+^ ions are represented as purple spheres. (*b*) An *F*_o_ − *F*_c_ OMIT map corresponding to the ligand (1.5σ). (*c*) Enlargement of the catalytic site showing the coordination of waters, ions and catalytic residues by the ligand. (*d*) Enlargement of the catalytic site of H1N1 FLUAV PA-N_ter_ in complex with baloxavir (PDB entry 6fs6).

Crystals of the LACV EndoN–baloxavir complex were obtained following the same procedure as for the L-742,001 complex in the presence of 5 m*M* MnCl_2_. The crystal diffracted to 2.2 Å resolution and was processed at 2.64 Å resolution in space group *P*6_1_22 with four copies of LACV EndoN in the asymmetric unit. The structure was then refined to an *R* factor and *R*_free_ of 0.21 and 0.25, respectively, with an average *B* factor of 59 Å^2^ [Fig. 4 ▸(*a*)]. The initial refinement after the molecular-replacement step revealed four *F*_o_ − *F*_c_ extra densities in the active site of each molecule. As expected, two dinuclear Mn^2+^ centres and baloxavir exhibit clear electron densities [Fig. 4 ▸(*b*)]. The baloxavir structure can be decomposed into two linked tricyclic scaffolds, a metal-chelating scaffold comprising an hydroxypyridotriazinedione motif and a specificity scaffold comprising a dibenzothiepine moiety. Mn1 is present in the active site of all four molecules and is coordinated by the side chains of His34, Asp79 and Asp92 and the carboxylic group of the Tyr93 main chain. Its strong coordination is reflected by an average *B* factor of 52 Å^2^, while the second manganese, Mn2, has an average *B* factor of 80 Å^2^. Three molecules of baloxavir were placed (chains *A*, *C* and *D*). The *F*_o_ − *F*_c_ density of chain *B* has a lower quality, making it impossible to locate baloxavir correctly. This is explained by the double conformation of the Tyr49–Phe55 loop containing the catalytic Asp52 residue and the high *B* factor of Mn2. One conformation allows the side chain of Asp52 to coordinate the manganese ion Mn2, and the other is in an open form that moves it away from the active site. The chelation of baloxavir, as observed for the chelating DKA motif of L-742,001, occurred via the three coplanar O atoms of the hydroxypyrido­triazinedione motif that bind Mn1 and Mn2; each metal ion displays octahedral coordination with proteic catalytic residues and hydration shell [Fig. 4 ▸(*c*)]. The chelation and specificity motifs of the ligand adopt the same conformations in the active sites of the three molecules. The dibenzothiepine moiety adopts a V shape and makes contact along the Met31 side chain, as for Ile38 in H1N1 FLUAV PA-N_ter_ [Fig. 4 ▸(*d*)].

The chelation and specificity motifs are positioned similarly in the two proteins, despite the absence of the N-terminal end of helix α2 carrying Tyr24 in the LACV EndoN active site, which is responsible for the locking of baloxavir into the H1N1 FLUAV PA-N_ter_ active site.

#### 3.2.3. Drug-design strategies

This study provides evidence that the metal chelators L-742,001 and baloxavir present an inhibitory effect on the LACV EndoN activity below the micromolar range. This result is supported by biophysical data as well as by two crystal structures. We can assume that ligands compete with the RNA substrate at the active site of LACV EndoN and are bound via metal chelation and specific amino-acid interactions. The challenge of designing inhibitors for an open cavity such as *Bunyavirales* EndoN lies in the fact that there is only a very small anchoring point to ensure the proper presentation of the inhibitor to its target. The use of a chelator in an open pocket should be considered more for its affinity (anchoring property) rather than for its chelating aspect (if the chelator is too strong then the ion will be depleted and loses its purpose). In addition to the chelating/anchoring motif, rational expansion of the molecule to achieve specificity for common EndoN structural features such as the conserved amino-terminal α-helix should be considered. As the flexibility of the *Bunyavirales* EndoN active site is responsible for the ligand-fitting mode, we suggest the design of bulky optimized inhibitors that should possess a more favourable binding free energy due to the gain in conformational entropy upon binding.

**Figure 5:**
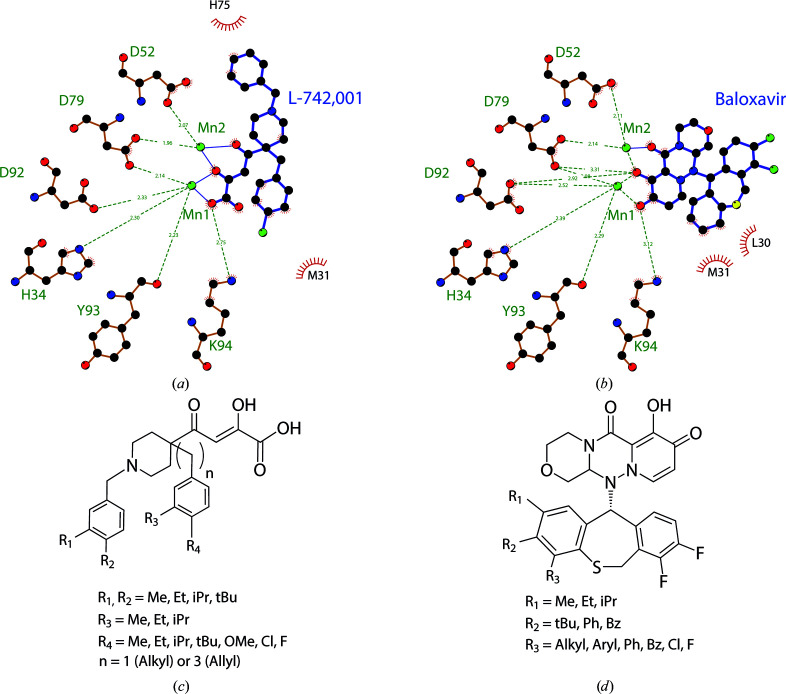
*LigPlot* and proposed structural modifications of the ligands L-742,001 and baloxavir. (*a*) and (*b*) show *LigPlot* representations of LACV EndoN in complex with L-742,001 and baloxavir, respectively. (*c*) and (*d*) represent the proposed modifications of L-742,001 and baloxavir, respectively.

Thanks to structural and *LigPlot* analysis, some hit-to-lead optimizations are proposed in Fig. 5 ▸. A potent drug-design strategy to improve LACV EndoN ligand binding is to penetrate farther into identified small pockets, forming favourable hydrophobic interactions. For the design of L-742,001 analogs [Figs. 5 ▸(*a*) and 5 ▸(*c*)], the benzyl ring facing His75 should be substituted with small alkyl groups in the *R*_2_ position to reinforce π-stacking interactions, and the same strategy should be proposed in the *R*_1_ position to extend the interactions with Ile76 and Leu112 delimitating a small hydrophobic cavity. There is still space to extend the *p*-chlorobenzyl arm with a vinyl chain to target Met31, to introduce small alkyl groups in the *R*_3_ position to face His34 and to substitute chlorine in the *R*_4_ position to increase the interactions with Leu30 and Arg33. It would be efficient to look for more buried interactions, in particular with folding towards the interior of the pocket to target Leu30 and Arg33.

For the design of baloxavir analogs [Figs. 5 ▸(*b*) and 5 ▸(*d*)], we speculate that modifying the benzylthiepine side at positions *R*_1_, *R*_2_ and *R*_3_ is a reasonable strategy. The *R*_1_ position facing His34 should be substituted by small alkyl groups to reinforce π-stacking interactions, the *R*_2_ position offers the possibility to introduce larger groups and aromatic rings to target inter­actions with Leu30 and Arg33, and in position *R*_3_ there is still more space to extend for buried interactions with Leu30 and Val27.

## 4. Conclusions

We report two crystallographic structures of LACV EndoN in complex with two divalent metal ions (Mn^2+^) and L-742,001 or baloxavir ligands. These structures, together with biophysical and biochemical data, provide strong evidence that both ligands compete with the RNA substrate at the active site of LACV EndoN and bind *via* metal chelation and specific amino-acid interactions. Based on these results, and the flexibility of the *Bunyavirales* EndoN active site responsible for the ligand-fitting mode, we suggest the design of bulky optimized inhibitors. In spite of the challenge of an open active site, this study has demonstrated the possibility of anchoring the molecule through a chelating motif and the importance of the knowledge of ligand-bound structures to guide the design of optimized inhibitors against the *Orthobunyavirus* endo­nuclease domain.

## Supplementary Material

PDB reference: N-terminal endonuclease domain of La Crosse virus L protein, complex with compound L-742,001, 7oa4

PDB reference: complex with baloxavir, 7plr

## Figures and Tables

**Table 1:** Data-collection and refinement statistics for the LACV EndoN domain complexed with ions and L-742,001 or baloxavir Values in parentheses are for the outer shell.

	LACV EndoN complexed with L-742,001	LACV EndoN complexed with baloxavir
PDB code	7oa4	7plr
Diffraction source	PROXIMA-2, SOLEIL	PROXIMA-1, SOLEIL
Wavelength (Å)	0.97563	0.97856
Resolution range (Å)	108.071–2.900 (2.975–2.900)	108.026–2.640 (2.709–2.640)
Space group	*P*6_1_22	*P*6_1_22
*a*, *b*, *c* (Å)	124.79, 124.79, 295.74	124.74, 124.74, 295.25
α, β, γ (°)	90, 90, 120	90, 90, 120
Molecules in asymmetric unit	4	4
Total No. of reflections	1231383 (126223)	1625854 (154013)
No. of unique reflections	31157 (3027)	40799 (3985)
Completeness (%)	99.94 (99.80)	99.95 (100.00)
Multiplicity	27.8 (27.09)	39.8 (39.10)
〈*I*/σ(*I*)〉	13.2 (2.2)	20.1 (2.2)
Overall *B* factor from Wilson plot (Å^2^)	52.78	52.83
*R*_p.i.m._	0.022 (0.089)	0.025 (0.190)
CC_1/2_	0.999 (0.980)	0.999 (0.912)
No. of reflections, working set	31073 (2145)	38758 (2821)
No. of reflections, test set	1542 (116)	2041 (145)
Final *R*_cryst_	0.197 (0.295)	0.214 (0.312)
Final *R*_free_	0.239 (0.351)	0.254 (0.337)
No. of non-H atoms		
Total	6386	6391
Protein	6142	6198
Ligand	142	130
Water	102	63
R.m.s. deviations		
Bond lengths (Å)	0.013	0.013
Angles (°)	1.63	1.62
Average *B* factors (Å^2^)		
Overall	56.56	59.52
Proteins	56.63	59.24
Ligands	64.12	76.97
Solvent	41.96	51.73
Ramachandran plot		
Most favoured (%)	96.29	97.80
Allowed (%)	3.43	2.06

**Table 2:** Biochemical and biophysical determinants of DPBA, L-742,001 and baloxavir against LACV EndoN

	Inhibition	Stability	Affinity
Compounds	IC_50_ (µ*M*)	Δ*T*_m_ (°C)	*K*_d_ (µ*M*)
DPBA	0.99 ± 0.06	+6.98 ± 0.25	1.35 ± 0.25
L-742,001	0.65 ± 0.03	+11.23 ± 0.15	0.614 ± 0.098
Baloxavir	0.34 ± 0.02	+12.72 ± 0.18	0.019 ± 0.006
